# Eleven quick tips to unlock the power of in vivo data science

**DOI:** 10.1371/journal.pcbi.1012947

**Published:** 2025-04-17

**Authors:** Troy J. Kieran, Taronna R. Maines, Jessica A. Belser

**Affiliations:** Influenza Division, Centers for Disease Control and Prevention, Atlanta, GeorgiaUnited States of America; Montreal, Quebec, CANADA

## Introduction

In vivo experimentation is an essential component of biological research. While these experiments enable scientific advances that benefit human and animal health, ethical and welfare considerations in the responsible use of research animals must be acknowledged and addressed [1]. Ethical principles of in vivo research are guided by the three R’s (reduction, refinement, replacement) [2], and compel investigators to reduce animal distress, use fewer animals, and pursue non-animal alternatives. Data sharing of in vivo results and data science incorporating these findings are underutilized areas that support these goals [3].

Recent efforts have sought to improve reporting of in vivo data in scientific publications (e.g., the ARRIVE guidelines [4]) and facilitate sharing and access of all data types (e.g., the FAIR guiding principles [5]). While these documents highlight that in vivo data can benefit from increased consistency and public availability, they lack specific guidance on organizing and presenting results for data science. Furthermore, FAIR guidelines address data sharing broadly, but not specifically for in vivo work, which may warrant specific considerations. Despite the complexity of in vivo experiments across various life science applications [6], commonalities exist in structuring these data for data science irrespective of experimental design.

**Fig 1:**
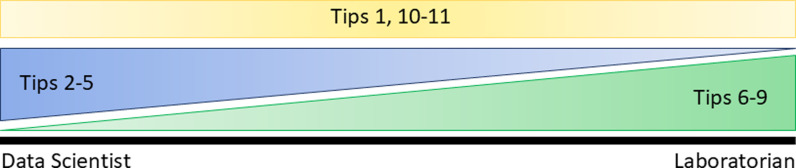
Utility of tips by subject matter expertise. Tips presented in this article fall under the primary purview of data scientists (Tips 2–5, supported by considerations presented in Table 1) or laboratorians (Tips 6–9, supported by considerations presented in Fig 2), though overlap may be high depending on subject matter expertise. Other tips span the entirety of the project (Tips 1, 10–11) and are applicable to all investigators from diverse backgrounds.

Increasing use of data science applications with in vivo data highlights the importance of compilation and sharing data practices [7–10]. Aggregating in vivo data can aid robust data management plans [11] and adherence to NIH data-sharing policies [12]. These efforts improve data organization and retention while facilitating new insights, if aggregation allows for responsible interpretation of results. This article builds upon best practices to prepare independently generated in vivo data for data science use, with an emphasis on subsequent data release. The following tips cater to a diverse audience of both data scientists and laboratorians (Fig 1), acknowledging the variability inherent in studies conducted and data generated in vivo.

**Table 1:** Representative examples of data types typical of in vivo experiments and when combining variables may be appropriate or problematic.

Broad category	Categorical and Numerical examples within category	Specific example	Example of combining appropriate	Example of combining problematic
Demographic	Categoric: species/strain/substrain, sex	Dataset includes both male and female animals	If prior studies support no sex-related differences in outcome variable [25]	If animal sex influences parameters (e.g., drug pharmacokinetics) of interest [30]
	Numeric: age, morphological quantifications			
Physiological	Categoric: developmental stage, previous procedure history	Dataset includes animals of varying pre-treatment weights and temperatures	If weight variances are normalized as a percentage change from baseline [17]	If temperature was quantified differently between studies (e.g., transponder vs use of rectal thermometer) [31]
	Numeric: body temperature, blood pressure, weight, biochemical/metabolic levels			
Behavioral	Categoric: action/mobility, verbal/cognitive cues	Dataset includes records of daily activity scoring	If the same scoring rubric was employed consistently across all studies/animals	If different rubrics or durations of observation were employed across studies [32]
	Numeric: activity score			
Environmental	Categoric: food source, enrichment provided	Dataset includes experiments performed year-round	If facilities have air control and lighting levels that can provide consistent environmental levels across seasons	If biological rhythms modulate outcome variables [33]
	Numeric: room temperature/humidity levels, time of day			
Pharmacological procedures	Categoric: drug formulation, drug lot, site and route of administration, frequency of administration, vehicle, or carrier solution	Dataset includes animals treated with multiple drugs with similar mechanisms of action	If outcome variable (e.g., pathogen load reduction, serum analyte level) is similarly affected independent of specific drug	If different drugs in dataset are differentially susceptible or resistant to the challenge pathogen
	Numeric: dose, volume, concentration			
Pathogen infection	Categoric: pathogen strain, passage history, site/route of administration, quantification method, timing/frequency of infection	Dataset includes animals inoculated with different doses of virus	If concentrations of virus employed lead to a course of infection yielding comparable outcome variables [7]	If concentrations of virus employed yield different results of outcome variable [34]
	Numeric: dose, volume			
Experimental results	Categoric: absence or presence of clinical signs	Dataset includes serially collected specimens on different schedules	If peak measurement spans full data collection time points of all included experiments	If measurement requires sampling on a specific day/time and combined results are inconsistent [35]
	Numeric: viral titers, weight change, quantifiable experimental measurements			

### Tip 1. Know the essentials of data science: Unleash potential

Compiling and analyzing data from in vivo experimentation is similar to working with other data types. Most best practices for data science apply to in vivo generated data. As with any data science endeavor, build your dataset in a suitable digital format [13], and follow best practices for spreadsheet organization [14]. We recommend comma-separated values (CSV) files for numerical and categorical data, ensuring compatibility with statistical and machine learning tools.

Depending on the type and scope of in vivo data aggregated, some considerations will require dedicated attention, as discussed throughout the article. The variety of data sources from in vivo experimentation may necessitate different data structures (e.g., separate tables with metadata on experimental treatments, pathogens, experimental datapoints, etc.). We recommend generating independent datasets with consistent naming conventions to facilitate merging during subsequent analyses.

Furthermore, the initial aggregation of the dataset does not determine its final structure. Specify how each row is populated (including unique animal tag numbers, full pathogen strain designations, experiment dates, researcher names, relevant publications, etc.). These metadata can be modified before release to address privacy and/or security concerns (e.g., anonymizing IDs or removing sensitive information). However, including metadata at the onset avoids duplicating records and facilitates rapid identification of specific entries. The benefits of aggregating in vivo data spanning numerous independent studies include identifying patterns that may be missed in individual studies, such as increased effect sizes by pooling data from larger experimental groups or uncovering unrecognized trends through correlation analyses across numerical variables.

### Tip 2. Be judicious when considering aggregating data: Selective inclusion

Animal experiments are designed to meet defined study objectives, with manipulation (often deliberately) of variables to assess pre-determined hypotheses [15]. Retrospective data science objectives may differ from the original in vivo study goals. Therefore, keeping an open mind when identifying studies appropriate for aggregation, even those with different purposes, can be beneficial.

The easiest studies to aggregate are often those conducted similarly, as these will likely have fewer differing variables that may introduce variability in analyses. However, casting a wide net can be beneficial if all varying parameters are captured in the dataset. Inclusion and exclusion criteria for aggregation may differ from those governing the initial experiment, depending on the outcome variable(s) evaluated. For example, an animal with a malfunctioning biometric chip might be excluded from one analysis for missing critical data but included in a dataset if a different outcome variable (such as a survival outcome not requiring data from the chip) is the analysis objective.

### Tip 3. Determine experimental and metadata to integrate: Picture building

After identifying the scope of targeted experiments, the next decision is determining which experimental variables to include when compiling results. To minimize revisiting source files, it’s advisable to include as many datapoints as possible during initial dataset building. Frequently captured variables will likely include those consistently collected across multiple studies and least affected by protocol-, facilities-, or personnel variability. In vivo experiments conducted over long periods with varying protocols should include appropriate metadata to enable filtering by similar experimental designs. Consider all data important, even if not used in the original analysis. Table 1 highlights broad categories of data types to capture, though inclusion of additional parameters specific to the aggregated experiments are encouraged.

The specific variables included in your dataset(s) will vary depending on the nature of the aggregated data and will likely encompass a range of numeric and categoric parameters (including but not limited to those in Table 1). Domain expertise is crucial; consider excluding datapoints subject to biases exhibiting uncontrollable variability, such as unblinded observational parameters prone to laboratorian biases. When uncertain, start with variables with the most specificity to allow for subsequent manipulation within the dataset without returning to multiple source files.

### Tip 4. Avoid transformation prior to raw data aggregation: Be patient

Aggregated files will include a mix of data types across multiple input files, encapsulating numerous datapoints, experimental observations, and substantial metadata. A central challenge in aggregating in vivo data is distilling complex, often serially-collected data into discrete values for column entry [16]. Compile experimental variables in a format that permits wide downstream mutability, as there are multiple ways serially collected data may be summarized.

Often, in vivo experiments collect raw observations (e.g., animal weights in grams, concentration of target per unit volume) before normalizing them for analyses (e.g., percentage change in weight over time, concentration fold change over mock-treated animals) [17]. Unless you have a clear plan for subsequent analyses, consider entering both non-normalized and normalized data from the start (for example, actual gram weights of animals in addition to normalized percentages of weight variability over time) as separate columns. This approach makes it easier to access non-normalized data later for analyses or inquiries about baseline values.

Many variables from in vivo experimentation can be captured in both numeric and categoric entries. For example, animal age could be reported numerically (months at treatment) or categorically (‘juvenile’ or ‘adult’), or an experimental readout frequency could be numeric (per-experiment percentage) or categoric (‘high’ or ‘low’ outcome). As shown in Table 1, a wide range of categories likely aggregated during compilation can span both considerations. Entering data with the highest granularity possible (e.g., including numeric values that can be classified categorically during data cleaning) will allow for greater manipulation of data across both classifications when feasible.

### Tip 5. Use the smallest experimental unit and sample identifiers: Puzzle pieces

**Fig 2:**
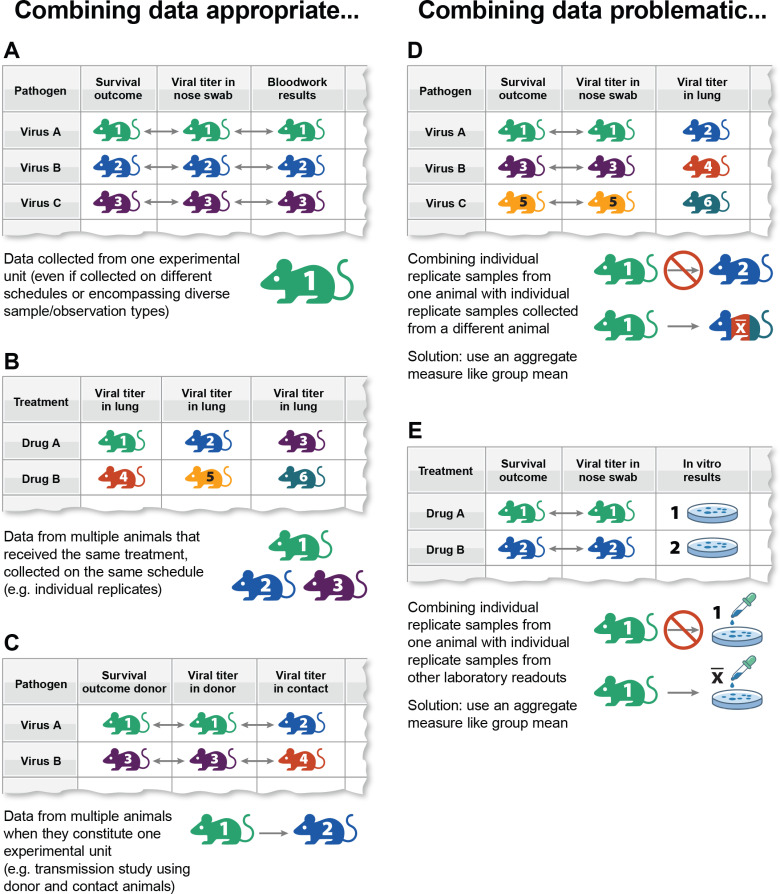
Graphical representation of data type examples typical of in vivo experiments and when combining variables may be appropriate (A–C) or problematic (D, E). Experimental units are designated by row. Individual animal/observation data are designated by color and number. *x̅*, mean value.

We emphasize the importance of a unique identifier for each experimental unit in the dataset, defined as “the biological entity subject to an intervention independently of all units” [4]. When aggregating in vivo data, each dataset row often encapsulates all data from a unique animal (Fig 2A). However, experimental units may also be subsets of data from one animal (e.g., if two different experiments are performed with the same animal at different times) or span multiple animals (e.g., pathogen transmission assessments linking donor and contact animals on the same row). We recommend entering data at the smallest experimental unit possible (e.g., per-animal) even if group means or other summary measures are the ultimate analysis goal. This allows for future dataset expansion and easy mean value updates using code. In contrast, aggregating initial mean values can complicate dataset modification, reduce reproducibility, and decrease statistical power due to a smaller overall sample size.

### Tip 6. Consider linking observations and experiments when appropriate: Connect dots

In vivo experiments often use multiple animal groups to achieve experimental outcomes. This can include assessing treatment or infection effects over time in one group (e.g., animal survival during acute infection) with a diversity of datapoints (e.g., viral titers or histopathological investigations) obtained from another group. In another setup, an inoculated donor animal is placed near a contact animal to assess pathogen spread. Linking data (placing datapoints on the same row) can be very insightful, but careful entry is essential for responsible interpretation.

As discussed, multiple variables may be in the same row when sourced from one animal from one experiment, as all sample types derive from the same experimental unit (Fig 2A). Multiple animals should only link to the same row if there is a true per-animal association. For example, individual replicates from multiple animals may be included in one row if all were treated identically and the reported variable was collected uniformly (Fig 2B). If multiple animals encapsulate the same experimental unit (e.g., a 1:1 donor:contact ratio transmission experiment), then data from both may also be included in the same row (Fig 2C).

However, linking datapoints across rows should be avoided in several situations. Serially collected samples from one group should not link on a per-animal basis with terminally collected samples from another group (Fig 2D), because these animals do not represent the same experimental unit. These data may still link in subsequent analyses, such as at a mean level (e.g., pairing an experimental unit to an aggregate of other experimental units).

### Tip 7. Expand analyses to incorporate non-in vivo experiments when warranted: Beyond in vivo

In vivo experiments are typically preceded or conducted alongside substantial in silico, in vitro, and other confirmatory work; linking these to animal datasets can be useful. Supporting datatypes might include pathogen molecular sequence data, ex vivo culture data, pharmacokinetic data of treatments administered, baseline culture results from pre-experimental manipulations, and environmental parameters, among others. Aggregating in vivo-generated results with supporting experimental data can pose challenges [16], and may be best compiled as independent datasets for later merging with aggregated in vivo data. Expanding experiment scopes for data science beyond in vivo generated data may help identify properties that refine the experimental model [18]. However, caution is needed when linking these data to per-animal results. Consider the experimental unit of the supporting data and avoid linking individual replicates of a non-in vivo study with individual in vivo replicates (Fig 2E).

### Tip 8. Address missing data with care: Mind the gap

Handling missing data is a common problem in data science [19]. Datapoints can be missing due to skipped observations, equipment malfunctions, or other scenarios, and differential classification of missing values is crucial. For example, if an animal requires humane euthanasia during an experiment, subsequent data collections will be ‘missing’. These events should be distinguished from cases where animals survived but experience sporadic missing datapoints for unrelated reasons (e.g., a malfunctioning biometric chip). Additionally, specimens that fall below a predetermined limit of detection during quantification may require separate identifiers in the dataset, along with a column specifying the limit of each assay. When aggregating experiments, similar samples might follow varied collection schemes (e.g., every-other-day collection schedules, but with different studies starting on day 1 or day 2). This variability could result in seemingly “missing” values despite complete sample collection, necessitating additional columns in the raw aggregated data to differentiate these samples from truly “missing” datapoints. Whether these data can be condensed into more generic “first” and “second” day data points is a careful decision based on what is appropriate for the data and downstream analyses.

### Tip 9. Be mindful of limitations: Push boundaries

In vivo-generated data may possess limitations not present in other lab analyses. While some issues, like sample size considerations, can be managed through careful interpretation, group sizes must balance statistical power with ethical use of animals [15]. Consequently, some analyses may be underpowered depending on the outcome variable [20, 21]. One way to address this is by combining experimental variables across studies that do not meaningfully affect the outcome. Table 1 provides examples of possible ways to achieve this, though the decision to combine will depend on domain expertise and validation experiments to avoid introducing inadvertent variability into the analysis. Clearly disclose any grouping of discrete values when describing the analysis methodology.

However, specific limitations exist in in vivo experiments where reconciliation may not be possible. For example, anesthesia requirements may extend time intervals between serially collected samples [22], hindering analyses which require frequent datapoints within defined periods. Outlier data points are not uncommon in studies conducted in vivo, attributed to technical errors, biological variation, or other reasons, warranting dedicated consideration for inclusion or exclusion [23]. Differences in phenotypic distributions may necessitate appropriate statistical or analytic approaches [24].

### Tip 10. Think about integrating externally generated data: Expand horizons

Depending on the experimental protocol, aggregated data may be entirely internal or from multiple external collaborators. Using externally generated data can increase sample size and rigor but may introduce variability due to confounding factors [25]. Domain expertise is key when balancing larger sample size against diversity, considering potential data variability or noise. External data incorporation is best for groups with similar experimental protocols, or to contextualize relevant metadata (non-in vivo generated, as discussed above). Despite challenges, these approaches can yield rigorous results by demonstrating that findings are reproducible across different research groups despite biological variation [24].

Utilizing existing publicly available data alongside your data can be important for validating analyses [7,26]. Externally generated data should generally capture similar inclusion/exclusion criteria as internal datasets to minimize confounders in subsequent analyses. However, preparing externally generated in vivo data for inclusion may require specific considerations, as with all biological data [27].

### Tip 11. Collaborate, share, and utilize your aggregated data: Maximize value

We encourage researchers to share data from standardized laboratory experiments by uploading it to public repositories, such as NCBI, Zenodo, Dryad, and Figshare. This ensures the dataset receives a persistent identifier (DOI) for easy citation and discovery. Alongside the dataset, provide relevant code for reproducing data compilation or variable modification to promote transparency and reproducibility. Include detailed metadata covering experimental design, laboratory protocols, and data analysis procedures. If applicable, identify peer-reviewed manuscripts that utilized the data and cross-reference this information with the dataset. Consider submitting papers to journals that publish Data Notes or Data Descriptors alongside primary manuscripts to highlight the data’s generation, uses, strengths, and limitations [28]. No dataset is too small for a detailed description. Foster collaborations with bioinformaticians or data scientists if you are a lab scientist and vice versa with laboratorians experienced in biological subject matter and in vivo data generation. Engaging these experts can clarify requirements for statistical techniques or machine learning methods suited to your biological data and identify appropriate lab questions for in vivo experiments. Participate in conferences, workshops, or online forums on the other side of the lab/analysis divide, leading to valuable engagement and potential collaborations.

## Conclusions

The use of animals in life sciences and humanities research dates to antiquity [29]. Essential improvements in animal welfare for these studies have significantly enhanced quality, rigor, and ethical use. Similar investments are needed in leveraging data from these studies in a data science context. However, aggregation and analyses must be performed with the same attention to detail as the experiment itself. As discussed, in vivo data may pose challenges for robust data science work but can provide meaningful insights towards improved research objectives and support best practices for responsible animal use in research settings.
